# Our experience with 80 cases of SARS-CoV-2-*Clostridioides difficile* co-infection: An observational study

**DOI:** 10.1097/MD.0000000000029823

**Published:** 2022-07-08

**Authors:** Victoria Birlutiu, Elena Simona Dobritoiu, Claudia Daniela Lupu, Claudiu Herteliu, Rares Mircea Birlutiu, Dan Dragomirescu, Andreea Vorovenci

**Affiliations:** a Faculty of Medicine Sibiu, Lucian Blaga University of Sibiu, Academic Emergency Hospital Sibiu—Infectious Diseases Clinic, Sibiu, Romania. Sibiu, Romania; b Bucharest University of Economic Studies, London Southbank University, Bucharest Romania, Bucharest, Romania; c Lucian Blaga University of Sibiu, FOISOR Clinical Hospital of Orthopedics, Traumatology, and Osteoarticular TB Bucharest, Sibiu, Romania; d Bucharest University of Economic Studies, Bucharest, Romania.

**Keywords:** co-infection, *Clostridioides difficile*, COVID-19, outcome, risk factors, SARS-CoV-2 infection

## Abstract

Beside the changes in the gut microbiota in context of severe acute respiratory syndrome coronavirus 2 (SARS-CoV-2) infection, the increased use of high-risk broad-spectrum antibiotics during the actual pandemic raises concerns about a possible increase of *Clostridioides difficile* infections (CDIs).

We retrospectively analyzed 80 consecutive patients, with SARS-CoV-2 pneumonia and CDI.

The mean length of hospitalization was 19.63 days. The mean time of the onset of the digestive symptoms related to CDI was 5.16 days. Patients with an onset of the digestive symptoms from hospital admission have a significantly lower median length in hospital stay. The recovered patients present a statistically significant decreased median age. coronavirus disease 2019 (COVID-19) cured patients present CDI symptoms much earlier than the deceased patients, when comparing the median days before the occurrence of any digestive symptoms regarding CDI. Among the patients that prior to their hospitalization for COVID-19 were exposed to antibiotics, 54.7% presented CDI digestive symptoms during their hospitalization and 65.6% had a severe or critical COVID-19 form.

Although the incidence of CDI in the pandemic is lower compared to the period before the pandemic, the severity of cases and the death rate increased. In the actual setting clinicians need to be aware of possible CDI and SARS-CoV-2 co-infection.

## 1. Introduction

The coronavirus disease 2019 (COVID-19) pandemic, which emerged in early December 2019 in Wuhan (China), is related to the severe acute respiratory syndrome coronavirus 2 (SARS-CoV-2), a strain of the Coronaviridae family, Orthocoronavirinae subfamily, and betacoronavirus (betaCoV) family.^[1]^ Due to the interhuman transmission of SARS-CoV-2 that allowed the infection, at the time of the writing (September 18, 2021) of this manuscript, the pandemic rapidly spread and caused 227,750,462 (infected) patients worldwide, resulting in 4681,306 deaths.^[2]^ COVID-19 predominantly includes pulmonary and cardiovascular symptoms; however, <10% of cases also include gastrointestinal events, including abdominal pain, diarrhea, and vomiting.^[3]^ Also, the neurotropic properties and the cutaneous manifestations of SARS-CoV-2 are increasingly identified.^[4–7]^

The increased use of high-risk broad-spectrum antibiotics during the actual SARS-CoV-2 pandemic raises concerns about a possible increase of *Clostridioides difficile* infections (CDIs), especially in the elderly and in the long-term care facilities residents’ population. *C. difficile*, a multiresistant pathogen, is the leading cause of diarrhea in healthcare settings, associated with antibiotic treatments, which presents a rate high of morbidity and mortality.^[8,9]^

At our institution, we found an increased in the use of antimicrobials overall and in high-risk CDI antibiotics beginning with March 2020.

In this setting, the Academic Emergency Hospital Sibiu, Romania, is involved from the beginning of this pandemic in the management of COVID-19 patients. This retrospective analysis describes the clinical characteristics, laboratory data, treatment, and the clinical outcome of our patients with laboratory-confirmed SARS-CoV-2 and CDI admitted into our hospital.

## 2. Materials and methods

A single-center observational cohort ongoing study on SARS-CoV-2 infected patients that associated with CDI is conducted in the Academic Emergency Hospital Sibiu, Romania, a county hospital with 1054 beds, dedicated for the treatment of COVID-19 patients from the beginning of this pandemic. In these analyses, we retrospectively analyzed 80 consecutive patients (36 female and 44 male patients), admitted to our hospital from April 1, 2020, until December 31, 2020, all with confirmed moderate or severe SARS-CoV-2 pneumonia (by real-time reverse transcriptase–polymerase chain reaction from nasal and pharyngeal swabs) that associated or not multiple systems organ failure, and CDI (by enzyme immuno assay for glutamate dehydrogenase associated with enzyme immuno assay to detect toxins A and B in stool samples). We used the Chinese CDC criteria for assessment of the pneumonia form, severe pneumonia was defined as: dyspnea, respiratory rate over 30/min, SpO_2_ below 93%, PaO_2_/FiO_2_ ratio <300, increase in the size of lung lesions by over 50% in the last 24 to 48 hours. Critically ill patients were defined as patients that were presenting respiratory distress, septic shock, and/or multiple organ dysfunction syndrome.^[1,10]^

The primary objectives were to assess the risk for developing CDI co-infection, the complications rate, and the effectiveness of the therapeutical management. The secondary objective was to assess the clinical and baseline characteristics and evolution of the patients.

All patients were treated by the standard of care according to the national guidelines. All patients diagnosed with sepsis, as well as patients with respiratory bacterial infections, received appropriate antibiotic therapy after a bacterial strain was identified (VITEK 2 Compact analyzer bioMérieux, Marcy-l’Étoile, France). The minimal inhibitory concentrations were assessed according to the EUCAST breakpoints.

Patient outcome and treatment effectiveness were assessed by clinical evolution and biologic markers.

Detailed information was abstracted from the medical records of the patients using a standardized collection form. All data were available for all the enrolled patients. The statistical analysis was performed using the IBM SPSS Statistics version 28 software. The analysis of categorical variables expressed as counts and percentage was performed using Chi-square and Fisher exact tests for investigating whether there is evidence for identifying differences between groups. Continuous variables were described as means (standard deviation) and medians (interquartile range). The assessment of differences in distributions between groups was performed using the Mann–Whitney nonparametrical test. A value of *P* ≤.05 was considered significant.

Patient follow-up ended at discharge (cured or deceased). Long-term prospective follow-up of our COVID-19 patients unfortunately is not yet available; it will be reported at the end of another ongoing study.

The study was accepted by the Ethics Committee of the hospital, and they encouraged publishing the article.

## 3. Results

Demographic and main baseline and clinical characteristics of the 80 enrolled patients are shown in the continuous and discreet data analysis (Tables 1 and 2). Thirty-six female and 44 male patients were enrolled in the study, with a mean age of 65.91 years (ranged from 22–93 years, std. deviation 14.31 years). According to the COVID-19 pneumonia severity grade classification, 78 of the 80 enrolled patients (97.5%) were moderate or seriously ill requiring noninvasive ventilation or oxygen mask, and 2 cases (2.5%) were critically ill requiring invasive mechanical ventilation. The mean length of hospitalization was 19.63 days (ranged between 1 and 50 days, SD = 9.06). The mean time of the onset of the digestive symptoms related to CDI was 5.16 days (ranged between 0 and 26 days, SD = 6.66), 44 patients presented CDI related digestive symptoms from the admission into the hospital, and 24 patients developed digestive symptoms until the 12th day of hospitalization.

**Table 1:** Demographic, baseline, and clinical characteristics.

Variable		Statistic
Age, yrs	Mean (SD)	65.91 (14.31)
	Minimum	22
	Maximum	93
	Median (IQR)	69 (17)
	SD	14.31
	Distribution(%)	
	≤ 39	5
	40–59	23
	60–74	43
	75+	30
Length in hospital stay (d)	Mean (SD)	19.63 (9.06)
	Minimum	1
	Maximum	50
	Median (IQR)	17.5 (12)
	SD	9.06
	Distribution (%)	
	≤7	3.75
	8–14	33
	15–21	28
	22–28	19
	29–35	14
	36+	4
CDI time to digestive symptoms	Mean (SD)	5.16 (6.66)
	Minimum	0
	Maximum	26
	Median (IQR)	0 (10)
	SD	6.7
	Distribution(%)	
	≤0	55.84
	1–12	29
	13+	15.58

CDI = *Clostridioides difficile* infection; IQR = interquartile range.

**Table 2:** Demographic clinical characteristics.

Variable		Frequency, *n*	Frequency, *n* (%)
Gender	Male	36	45
	Female	44	55
	Total	80	100
Area of origin	Urban	64	80
	Rural	16	20
	Total	80	100
COVID-19 severity level	Moderate	32	40
	Severe	46	57.5
	Critical	2	2.5
	Total	80	100.0

COVID-19 = coronavirus disease 2019.

The analysis of the laboratory data from the time of admission into the hospital revealed the following: a mean C-reactive protein 189.96 (±483.48 mg/L, reference value <6 mg/L), mean Procalcitonin 3.98 (±8.13 ng/L, reference value <0.15 ng/mL), mean D-dimer serum concentration 3289.94 (±5866.44 ng/mL, reference value <250 ng/mL), mean serum ferritin level 876.68 (±868.14 ng/mL, reference value 6–159 ng/dL), mean neutrophil-to-lymphocyte ratio 13.74 (±13.34), mean white blood cell count 14,851.4 (±10,798.39 µL, reference value 4000–10,000 µL), mean erythrocyte sedimentation rate 46 (±30 mm/h, reference level 0–20 mm/h), and a mean fibrinogen 522.41 (±170.05 mg/dL, reference level 170–420 mg/dL). Using a Mann–Whitney U test to compare whether there is a difference between the laboratory data and the discharge status, time to digestive symptoms, COVID-19 disease form, previous hospitalization, or intensive care unit hospitalization, there were no statistically significant differences.

From the comorbidities point of view, 40 patients presented hypertension, 25 other cardiovascular diseases, chronic obstructive pulmonary disease 5 cases, type 2 diabetes mellitus 24 cases, obesity 19 cases, chronic kidney disease 8 cases, solid tumors 7 cases, hematological malignancy 2 cases, stroke 8 cases, other neurological disorders 9 cases, depressive syndrome 5 patients, other mental disorders 10 patients, digestive disorders (inflammatory bowel disease, cholecystectomy, other digestive disorders) 17 patients, endocrine disorders 6 patients, urological disorders 5 patients, and autoimmune diseases 2 patients.

Data in our study shown that 60 of the 80 cases (75%) progressed to a favorable outcome and 20 unfortunately toward death.

Continuous variables analysis is shown in Tables 3–5.

Of the 80 patients included in the analysis, there was no statistical difference between male and female patients, regarding age, length of hospital stay, or time to the occurrences of the digestive symptoms.

Patients with an onset of the digestive symptoms from hospital admission have a significantly lower median length in hospital stay than the patients with an onset of the digestive symptoms later, during hospitalization (15 vs 21, Sig. < 0.05). Another difference is shown in the age of the patients when analyzing their survival. The recovered patients present a statistically significant decreased median age than the patients that did not survive (67 vs 76, Sig. < 0.05, Mann–Whitney U test). COVID-19-cured patients are shown to present CDI symptoms much earlier than the deceased patients, when comparing the median days before the occurrence of any digestive symptoms regarding CDI (1 vs 8.5, Sig < 0.05).

The patients that presented a moderated form of COVID-19 have a significantly lower median age than the patients with a severe or critical form of COVID-19 (65.5 vs 71, Sig. < 0.05).

COVID-19 severe or critical patients present a significant increase in the median number of days until they presented CDI-associated digestive symptoms (0 vs 7, Sig. < 0.001).

The treatment of SARS-CoV-2 infection was performed following the local and national guidelines. SARS-CoV-2 infection treatment analysis is presented in Table 6. Treatment was initiated before hospitalization with azithromycin 500 mg on the first day, and then 250 mg/d for 4 days in 26 patients by the general practitioner. Thirty-eight patients received during hospitalization lopinavir/ritonavir 200/50 mg two tablets q12h for 10 days, 34 received with hydroxychloroquine, 200 mg q12h for 5 days. Fourteen patients received Remdesivir day 1 loading dose: 200 mg IV infused over 30 to 120 minutes, then 100 mg IV qday for 5 days, and for the patients that required invasive mechanical ventilation treatment was extended up to 10 days total. Nine hospitalized patients received Favipiravir 1.6 g q12h on day 1, followed by 600 mg q12h for a total duration of 7 to 14 days.

**Table 6:** SARS-CoV-2 infection treatment analysis.

		Lopinavir/Ritonavir		Sig.	Hydroxychloroquine		Sig.	Remdesivir		Sig.	Favipiravir		Sig.	Azithromycin		Sig.
	All, *N* = 80	No treat., *n* = 42	With treat., *n* = 38		No treat., *n* = 46	With treat., *n* = 34		No treat., *n* = 66	With treat., *n* = 14		No treat., *n* = 71	With treat., *n* = 9		No treat., *n* = 54	With treat., *n* = 26	
Discharge status																
Cured, *n* (%)	60 (75.0%)	30 (71.4%)	30 (78.9%)	0.438	33 (71.7%)	27 (79.4%)	0.433	52 (78.8%)	8 (57.1%)	0.102	54 (76.1%)	6 (66.7%)	0.684	38 (70.4%)	22 (84.6%)	0.168
Deceased, *n* (%)	20 (25.0%)	12 (28.6%)	8 (21.1%)		13 (28.3%)	7 (20.6%)		14 (21.2%)	6 (42.9%)		17 (23.9%)	3 (33.3%)		16 (29.6%)	4 (15.4%)	
Time to digestive symptoms																
From admission, *n* (%)	43 (53.8%)	24 (57.1%)	19 (50.0%)	0.522	26 (56.5%)	17 (50%)	0.563	41 (62.1%)	2 (14.3%)	**0.001**	40 (56.3%)	3 (33.3%)	0.290	31 (57.4%)	12 (46.2%)	0.344
During hospitalization, *n* (%)	37 (46.3%)	18 (42.9%)	19 (50.0%)		20 (43.5%)	17 (50%)		25 (37.9%)	12 (87.7%)		31 (43.7%)	6 (66.7%)		23 (42.6%)	14 (53.8%)	
COVID-19 severity level																
Medium, *n* (%)	32 (40.0%)	17 (40.5%)	15 (39.5%)	0.927	20 (43.5%)	12 (35.3%)	0.460	31 (47.0%)	1 (7.1%)	0.006	27 (38%)	5 (55.6%)	0.472	21 (38.9%)	11 (42.3%)	0.770
Severe/critical, *n* (%)	48 (60.0%)	25 (59.5%)	23 (60.5%)		26 (56.5%)	22 (64.7%)		35 (53.0%)	13 (92.9%)		44 (62%)	4 (44.4%)		33 (61.1%)	15 (57.7%)	

The bold numbers correspond to/are the significance values.

COVID-19 = coronavirus disease 2019.

There is a statistically significant association between the time that the digestive symptoms debuted and the likelihood of reporting that the patients had a COVID-19 treatment with Remdesivir to evaluate Tests of Independence when using a cross-tabulation. It can be observed that a significantly higher percentage of the patients who received Remdesivir debuted the CDI-associated symptoms during their hospitalization, versus the patients admitted in the hospital already showing digestive symptoms (87.7% vs 14.3%, Chi-square test, *P* < .001).

CDI treatment analysis is presented in Table 6. Twenty-eight patients received oral vancomycin, 6 patients received intravenous metronidazole, 44 patients were under treatment with oral vancomycin associated with intravenous metronidazole, 36 patients received intravenous tigecycline, and 17 patients received oral rifaximin. Statistically significant associations can be observed between the onset time of the digestive symptoms and the likelihood of reporting that the patients received a CDI treatment with vancomycin. A higher percentage of patients who received vancomycin presented digestive symptoms after their admission into the hospital. A higher percentage of patients that were cured did not receive tigecycline treatment, the differences between groups being statistically significant, using the Chi-Square test (Sig. < 0.05) (Table 7).

**Table 7:** *Clostridioides difficile* infection treatment analysis.

		Vancomycin			Metronidazole			Vancomycin+Metronidazole			Tigecycline			Rifaximin		
	All, *N* = 80	No treatment, *n* = 52	With treatment, *n* = 28	Sig.	No treatment, *n* = 74	With treatment, *n* = 6	Sig.	No treatment, *n* =36	With treatment, *n* = 44	Sig.	No treatment, *n* = 45	With treatment, *n* = 35	Sig.	No treatment, *n* = 63	With treatment, *n* = 17	Sig.
Discharge status																
Cured, *n* (%)	60 (75%)	36 (69.2%)	24 (85.7%)	0.104	56 (75.7%)	4 (66.7%)	0.637	28 (77.8%)	32 (72.7%)	0.604	38 (84.4%)	22 (62.9%)	0.027	47 (74.6%)	13 (76.5%)	1.000
Deceased, *n* (%)	20 (25%)	16 (30.8%)	4 (14.3%)		18 (24.3%)	2 (33.3%)		8 (22.20%)	12 (27.3%)		7 (15.6%)	13 (37.1%)		16 (25.4%)	4 (23.5%)	
Time to digestive symptoms																
From admission, n (%)	43 (53.8%)	33 (63.5%)	10 (35.7%)	0.018	41 (55.4%)	2 (33.3%)	0.407	13 (36.1%)	30 (68.2%)	0.004	25 (55.6%)	18 (51.4%)	0.713	34 (54.0%)	9 (52.9%)	0.940
During hospitalization, *n* (%)	37 (43.7%)	19 (36.5%)	18 (64.3%)		33 (44.6%)	4 (66.7%)		23 (63.9%)	14 (31.8%)		20 (44.4%)	17 (48.6%)		29 (46%)	8 (47.1%)	
COVID-19 form																
Medium, *n* (%)	32 (40%)	22 (42.3%)	10 (35.7%)	0.566	30 (40.5%)	2 (33.3%)	1.000	12 (33.3%)	20 (45.5%)	0.271	21 (46.7%)	11 (31.4%)	0.168	26 (41.3%)	6 (35.3%)	0.655
Severe/critical, *n* (%)	48 (60%)	30 (57.7%)	18 (64.3%)		44 (59.5%)	4 (66.7%)		24 (66.7%)	24 (54.5%)		24 (53.3%)	24 (68.6%)		37 (58.7%)	11 (64.7%)	

COVID-19 = coronavirus disease 2019.

A comorbidities and therapeutical risk factors analysis is reported in Table 8.

**Table 8:** Comorbidities and therapeutical risk factors analysis.

		COVID-19 form		Sig.
		Medium, *n* (%)	Severe/critical, *n* (%)	
Time to digestive symptoms	All (*n* = 80)	32 (40%)	48 (60%)	**0.002**
From admission, *n* (%)	43 (53.75%)	24 (55.8%)	19 (44.2%)	
During hospitalization, *n* (%)	37 (46.25%)	8 (21.6%)	29 (78.4%)	
		Discharge status		
		Deceased, *n* (%)	Cured, *n* (%)	
Hematology disorders	All (*n* =80)	20 (25%)	60 (75%)	**0.06**
No, *n* (%)	78 (97.5%)	18 (23.1%)	60 (76.9%)	
Yes, *n* (%)	2 (2.5%)	2 (100%)	0 (0.00%)	
		Time to digestive symptoms		
		From admission, *n* (%)	During hospitalization, *n* (%)	
Endocrinology disorders	All (*n* = 80)	43	37	**0.028**
No, *n* (%)	74 (92.5%)	37 (50%)	37 (50%)	
Yes, *n* (%)	6 (7.5%)	6 (100%)	0 (0.00%)	
		Time to digestive symptoms		
		From admission, *n* (%)	During hospitalization, *n* (%)	
Proton pump inhibitors	All (*n* = 80)	43 (53.8%)	37 (46.3%)	**0.015**
No, *n* (%)	13 (16.3%)	11 (84.6%)	2 (15.4%)	
Yes, *n* (%)	67 (83.7%)	32 (47.8%)	35 (52.2%)	
		Time to digestive symptoms		
		From admission, *n* (%)	During hospitalization, *n* (%)	
Steroidal anti-inflammatory drugs	All (*n* = 80)	43 (53.8%)	37 (46.3%)	**0.008**
No, *n* (%)	17 (21.3%)	14 (82.4%)	3 (17.6%)	
Yes, *n* (%)	63 (78.8%)	29 (46.0%)	34 (54.0%)	
		Time to digestive symptoms		
		From admission, *n* (%)	During hospitalization, *n* (%)	
Antibiotics	All (*n* = 80)	43 (53.8%)	37 (46.3%)	**0.008**
No, *n* (%)	16 (20.0%)	14 (87.5%)	2 (12.5%)	
Yes, *n* (%)	64 (80%)	29 (45.3%)	35 (54.7%)	
		COVID-19 form		
		Medium, *n* (%)	Severe/critical, *n* (%)	
Antibiotics	All (*n* = 80)	32 (40%)	48 (60%)	**0.04**
No, *n* (%)	16 (20%)	10 (62.5%)	6 (37.5%)	
Yes, *n* (%)	64 (80%)	22 (34.4%)	42 (65.6%)	

The bold numbers correspond to/are the significance values. COVID-19 = coronavirus disease 2019.

*P* values <.05 significance level indicate that there is enough evidence to conclude that a relationship exists between the categorical variables included in the analysis. The COVID-19 severity level is related to the time that the digestive symptoms occurred (*P* < .05, Chi-square test of independence). For the other categorical variables (comorbidities), *P* values were >0.05 (95% confidence interval). Among the patients that debuted the digestive symptoms during hospitalization, 78.4% had a severe or critical COVID-19 form. Six patients presented a history of endocrinological disorders and all of them presented CDI digestive symptoms from their admission into the hospital. Among the patients that before their hospitalization for COVID-19-received proton pump inhibitors treatment, 52.2% presented CDI digestive symptoms during their hospitalization. Among the patients that before their hospitalization for COVID-19-received steroidal anti-inflammatory drugs, 54% presented CDI digestive symptoms during their hospitalization. Among the patients that before their hospitalization for COVID-19 were exposed to antibiotics, 54.7% presented CDI digestive symptoms during their hospitalization and 65.6% had a severe or critical COVID-19 form.

## 4. Discussion

CDI is associated with an impressive number of cases, annually around 500,000 cases are diagnosed in the US alone, resulting in 29,000 deaths,^[11]^ respectively, 152,905 cases in Europe, resulting in 8382 deaths annually.^[12]^

The most frequently affected risk group is the institutionalized elderly people who can be colonized with CD in a high percentage, up to 51%^[13]^ or, who present multiple comorbidities that require hospitalization, antibiotic therapy, proton pump inhibitors therapy, etc to which the emergence of highly virulent CD strains is added, such as BI/NAP1/027, strain that is responsible for increased morbidity and mortality from CDI. Among patients with symptomatic SARS-CoV-2 infection, 19% of them report digestive symptoms. Changes in the mucosa of the digestive tract, and the normal colonic microbiota, reduce the mechanisms of local defense against pathogenic flora, including CD,^[14]^ with the risk of a devastating diarrheal episode, especially in the elderly population.

In fact, there are changes in the gut microbiota similar to exposure to broad-spectrum antibiotics, in patients with SARS-CoV-2 infection, that present significant digestive symptoms.^[15]^ The significant inflammatory phenomena in the digestive tract, as well as the changes in the gut microbiota, with a decrease in its diversity, can also be attributed to angiotensin-converting enzyme 2 receptors and co-expression with the transmembrane protease (TMRPSS2) receptor.^[16]^ The severity of COVID-19 is associated with the abundance of *Coprobacillus* and *Clostridium* species (*ramosum* and *hathewayi*), *Str. infantins*, *Colinsella aerofaciens*, *Colinsella tanakaei*, and *Morganella morganii*.^[17,18]^

In cases with a reduced SARS-CoV-2 infectivity, there is an abundance in the gut microbiota of *Parabacteroides merdae*, *Bacteroides stercoris*, *Alistipes onderdonkii*, and *Lachnspiraceae bacterium*, butyrate type short-chain fatty acid-producing bacteria, bacterial strains that play an important role in local immunity, respectively, in maintaining local homeostasis. The fecal viral clearance may be associated with the presence of pathogenic bacterial strains, such as *Klebsiella pneumoniae*, *Citrobacter koseri*, and *Bifidobacterium dentium*.^[18]^

In patients with diabetes mellitus, high blood pressure, or cardiovascular disease, there are changes in the renin–angiotensin system, a decreased number of angiotensin-converting enzyme 2 receptors with consequences on the gut microbiota (dysbiosis). Most commonly, these comorbidities are associated with severe forms of SARS-CoV-2 infection.^[19–22]^ Also, in obese or elderly populations, inflammatory changes, and a decrease in the diversity of the gut microbiota are observed. In the latter, psychiatric therapy, and proton pump inhibitors therapy, significantly reduces intestinal biodiversity. In patients with SARS-CoV-2 infection, inflammatory changes in the gut as well as in the microbiota result in an altered gut-brain axis, favoring the appearance of mental disorders - depression, anxiety, panic attack, irregular sleep–wake rhythm, etc. Suetens et al^[23]^ report that from 2016 to 2017, for a total number of 310,755 patients from 28 EU/EEA countries, admitted in 1209 hospitals, at least 1 healthcare-associated infections was identified in 6.5% of cases. For 117,138 long-term care facilities residents from 23 EU/EEA countries, a healthcare-associated infection was registered in at least 3.9% of cases. Also, there is an alarming increase in antibiotic resistance to 31.6% in acute hospitals versus 28% in chronic care units.^[23]^

During the SARS-CoV-2 pandemic, with the increase in the use of antibiotic therapy, and corticosteroids, an increase in CDI cases was predictable, associating an increased risk of death of the hospitalized COVID-19 patients. From the very beginning of the pandemic, Sandhu et al^[3]^ noted only in the first 4 months of the pandemic, an increase in the incidence of CDI from 3.32/10,000 patients-day to 3.60/10,000 patients-day.

CDI represented 0.27% of the total hospitalizations in the last 5 years (2015–2019) in the Infectious Diseases Clinic of the Academic Emergency Hospital Sibiu, Romania. In 2020, 2351 COVID-19 patients were treated, 80 patients associated CDI, representing 3.40% of the cases.

Patients with hospitalized diarrhea performed favorably in a median hospitalization period of 15 days compared to those with late onset, in which the median hospitalization period was extended to 21 days, a statistically significant difference.

Patients with an onset of the digestive symptoms from hospital admission and a favorable outcome had a significantly lower median length in hospital stay (15 days) than the patients with an onset of the digestive symptoms later, during hospitalization, with a median length of stay of 21 days (Sig. < 0.05).

Luo et al^[24]^ retrospectively analyzed the cases of CDI before and during the SARS-CoV-2 pandemic, reported an increase in the period of hospitalization without noticing significant statistical differences in terms of gender, age, or ward (intensive care unit hospitalization), medical or surgical admission history. The authors also report the increased use of antibiotics that represent risk factors for CDI, respectively, third-generation cephalosporins, fluoroquinolones, or clindamycin.^[24]^ In our study, we found in addition to the increased period of hospitalization, and the use of third-generation cephalosporins, especially ceftazidime, (44.83% of cases) but also carbapenems (13.79% of cases), mainly in critical cases that required admission into the intensive care unit. 39.65% of cases associated digestive symptoms (diarrhea) only after the use of azithromycin with or without the use of other risk medications like steroidal anti-inflammatory drugs, or proton pump inhibitors. Moreover, the management of the patients with COVID-19 is a complex one, that requires the use of antiviral drugs (Favipiravir, Remdesivir), anticoagulants, anti-IL6, anti-IL1, therapy that is added to the therapy considered to be at risk for CDI, steroidal anti-inflammatory drugs, proton pump inhibitors, and antibiotics.^[25]^ Although in patients receiving Remdesivir, a more frequent association of CDI versus the patients admitted to the hospital already showing digestive symptoms (87.7% vs 14.3%) was found, the complex treatment of these cases should not be neglected, along with steroidal anti-inflammatory drugs, proton pump inhibitors, broad-spectrum antibiotic therapy, etc. Besides, the patients that before their hospitalization for COVID-19-received proton pump inhibitors treatment, 52.2% presented CDI digestive symptoms during their hospitalization. Among the patients that prior to their hospitalization for COVID-19-received steroidal anti-inflammatory drugs, 54% presented CDI digestive symptoms during their hospitalization. Among the patients that before their hospitalization for COVID-19 were exposed to antibiotics, 54.7% presented CDI digestive symptoms during their hospitalization and 65.6% had a severe or critical COVID-19 form.

The death rate in patients with COVID-19 that associated CDI was 25% (toxic megacolon, gastrointestinal sepsis) even if the deaths were not only attributable to CDI but also to the evolution of SARS-CoV-2 infection.

Therapeutical management of these cases was performed from the beginning with oral vancomycin and intravenous metronidazole. In the absence of a therapeutic response at 3 to 5 days after initiation of therapy or in the presence of other bacterial superinfections in the respiratory tract, tigecycline was associated. Twenty-eight patients received oral vancomycin, 6 patients received intravenous metronidazole, 44 patients were undertreatment with oral vancomycin associated with intravenous metronidazole, 36 patients received tigecycline, and 17 patients received rifaximin. If by 2019, cases of first relapse of CDI or with potential for a relapse of the CDI (patients undergoing chemotherapy, under treatment with steroidal anti-inflammatory drugs, patients with hematological malignancies, etc) benefited from a fecal microbiota transplant in 13.94% of cases, during the pandemic, for safety reasons for the patient (difficulty in determining the presence of SARS-CoV-2 in the stool), this method was used in only 2 cases.

From an economic point of view, the treatment of CDI before the SARS-CoV-2 pandemic represents 17.7% of the expenses of our Infectious Diseases Clinic, during the pandemic, CDI treatment management costs were only 0.34% of total expenditures.

In a Spanish study, it is reported the decrease of healthcare-associated infections CDI cases by 70%, which we also noticed, even if the use of broad-spectrum antibiotics has increased, due to the correct use of personal protective equipment by the medical staff, and the compliance with measures to isolate patients.^[26]^

In another study, although on a small number of patients with COVID-19 and CDI (9 cases), the mortality rate of 44.4% was reported to be the result of a combination of factors related to the age of patients, their comorbidities, not only associated with the 2 etiological agents.^[3]^

Studies regarding the CDI incidence during the pandemic report contradictory conclusions, like the one published by Laszkowska et al,^[27]^ where the authors consider that CDI is not an important etiology of diarrhea in patients with COVID-19, while Ferreira et al^[25]^ consider that the diagnosis of CDI during the pandemic can be underestimated, especially as the use of antibiotics has increased in this period, vigilance in investigation and diagnosis being required.

The main limitations of our study are the small sample size and the short period of observation.

## 5. Conclusions

In conclusion, although the incidence of CDI in the pandemic is lower compared to the period before the pandemic, the severity of cases and the death rate increased, according to our study, to 25%. In the actual setting of the COVID-19 pandemic and the extensive use of broad-spectrum antibiotics, clinicians need to be aware of possible CDI and SARS-CoV-2 co-infection. Local and national monitoring systems of CDI are mandatory to prevent the increase of CDI during the current COVID-19 pandemic.

**Table 3:** Continuous variables analysis.

		Gender		Sig.
	All, *N* = 80	Male, *n* = 36	Female, *n* = 44	
Age, yrs				
Mean (SD)	65.91 (14.30)	62.11 (16.44)	69.02 (11.57)	0.099
Median (IQR)	69 (17)	68 (26)	69.50 (16)	
Length in hospital stay				
Mean (SD)	16.93 (9.59)	19.83 (9.611)	19.45 (8.69)	0.884
Median (IQR)	17.50 (12)	16.50 (13)	18.00 (12)	
Time to digestive symptoms				
Mean (SD)	5.16 (6.66)	6.00 (7.29)	4.48 (6.10)	0.338
Median (IQR)	0.00 (10)	3.50 (12)	0.00 (10)	

**Table 4:** Continuous variables analysis.

		Time to digestive symptoms		Sig.
	All, *N* = 80	From admission, *n* = 43	Few days after admission, *n* = 37	
Age, yrs				
Mean (SD)	65.91 (14.30)	65.63 (13.72)	66.24 (15.14)	0.643
Median (IQR)	69 (17)	68.00 (17)	69.00 (23)	
Length in hospital stay				
Mean (SD)	16.93 (9.59)	17.21 (7.95)	22.43 (9.55)	**0.006**
Median (IQR)	17.50 (12)	15.00 (13)	21.00 (12)	
Time to digestive symptoms				
Mean (SD)	5.16 (6.66)	—	—	—
Median (IQR)	0.00 (10)	—	—	

The bold numbers correspond to/are the significance values.

COVID-19 = coronavirus disease 2019; IQR = interquartile range.

**Table 5:** Continuous variables analysis.

		Status		Sig.
	All, *N* = 80	Cured, *n* = 60	Deceased, *n* = 20	
Age, yrs				
Mean (SD)	65.91 (14.30)	63.08 (14.23)	74.40 (11.01)	**0.001**
Median (IQR)	69 (17)	67.00 (23)	76.00 (10)	
Length in hospital stay (days)				
Mean (SD)	16.93 (9.59)	19.40 (7.58)	20.30 (12.73)	0.833
Median (IQR)	17.50 (12)	17.50 (11)	17.00 (22)	
Time to digestive symptoms (d)				
Mean (SD)	5.16 (6.66)	4.22 (6.37)	8.00 (6.85)	**0.015**
Median (IQR)	0.00 (10)	1.00 (9)	8.50 (14)	
		**COVID-19 form**		**Sig.**
	**All, *N* = 80**	**Medium, *n* = 32**	**Severe/critical, *n* = 48**	
Age, yrs				
Mean (SD)	65.91 (14.30)	69.69 (16.53)	69.40 (11.52)	**0.016**
Median (IQR)	69 (17)	65.50 (22)	71.00 (15)	
Length in hospital stay (d)				
Mean (SD)	16.93 (9.59)	17 (5.87)	21.38 (10.36)	**0.058**
Median (IQR)	17.50 (12)	15.50 (9)	21.00 (16)	
Time to digestive symptoms (d)				
Mean (SD)	5.16 (6.66)	2.16 (3.96)	7.17 (7.34)	**<0.001**
Median (IQR)	0.00 (10)	0.00 (5)	7.00 (12)	

The bold numbers correspond to/are the significance values.

IQR = interquartile range.

## Author contributions

VB, RMB, AV, and DD contributed in equal parts. VB, CDL, and ESD designed the study and coordinated data collection. VB, CDL, and ESD were involved in providing the treatment for the patients and in collecting the data. AV and DD performed the statistical analysis of the data. VB, RMB, DD, CH, and AV were involved in the interpretation of data. RMB, VB, DD, and AV were involved in drafting the manuscript. All authors were involved in revising the manuscript. All authors read and approved the final version of the manuscript.
